# Classification of vascularized fibular flap hypertrophy based on X-ray evaluation

**DOI:** 10.5152/j.aott.2021.20206

**Published:** 2021-11-01

**Authors:** Tulgar Toros, Murat Kayalar, Kemal Özaksar, Tahir Sadık Sügün, Yusuf Gürbüz

**Affiliations:** Department of Orthopedics and Traumatology, Hand and Microsurgery & Orthopedics and Traumatology, EMOT Hospital, İzmir, Turkey

**Keywords:** Reconstruction, Vascularized fibular flap, Hypertrophy, Classification

## Abstract

**Objective:**

The aim of this study was to analyze and classify hypertrophy seen in vascularized fibula flaps used for reconstruction of tubular bone defects.

**Methods:**

Thirty-three patients who underwent a vascularized fibula flap for the reconstruction of massive bone defects of the upper or lower extremity long bones were retrospectively reviewed and included in this study. There were 24 lower extremities (21 tibial and 3 femoral) and 9 upper extremities (4 humeral, 2 radial and 3 ulnar) reconstructions in this series. The mean age was 32.7 (range= 10– 59) years. The mean length of bony defect following initial debridement was 10.3 (range= 4–25) cm. The fibula was inserted as a single strut in 29 patients, and as a double barrel construct in 4 patients. The degree of fibular hypertrophy was calculated based on anteroposterior (AP) and lateral X-ray measurements of fibular flaps at an average postoperative period of 52 months. The difference in thickness between the initial and final x- ray measurements were expressed as percentage of hypertrophy. The variances seen in this period were defined and classified.

**Results:**

When bony consolidation of the 33 cases were examined in detail, 4 different modes of flap hypertrophy were defined: type 0- absence of hypertrophy, type 1- limited hypertrophy, type 2- marked hypertrophy triggered by stress fracture, and type 3-massive hypertrophy enhanced by peripheral bone production.

**Conclusion:**

Fibular hypertrophy follows different modes based on vascularity of the flap, amount of stress imparted on the flap, site of reconstruction, and whether the periosteal sleeve is retained at the reconstruction site. Determination of these factors at the initial period may help the surgeons to predict the final hypertrophy that will be seen at the end of flap maturation

**Level of Evidence:**

Level IV, Therapeutic Study

## Introduction

Several studies have indicated that vascularized fibular flap hypertrophies occur under certain conditions when transferred to reconstruct a long-bone defect; however, the variations and mechanisms involved in this reshaping have not been identified or classified.^1,2^ This study aims to analyze and classify the flap hypertrophy seen in vascularized fibulas following transfer for reconstruction of tubular bone defects. The proposed classification system is based on X-ray findings and is intended to predict the vascularized fibular flap enlargement results seen in tubular bone reconstructions.

## Materials and Methods

This retrospective study was performed on a series of 33 segmental bone reconstructions of the upper and lower extremities performed between 1993 and 2016; 31 of these patients showed proven viability of the fibular flap and two had non-viable flaps due to early complications. Institutional review board approval was obtained from the hospital at which this study was conducted, and written informed consent was obtained from the patients who participated in this study.

Detailed numeric data from the involved patients are presented in Tables 1 and 2. There are 24 lower extremity (21 tibial and 3 femoral) and 9 upper extremity (4 humeral, 2 radial, and 3 ulnar) reconstructions in this series. The average age of patients at the time of the index operation was 32.7 (range: 10-59) and the length of bony defects following initial debridement averaged 10.3 cm (range: 4-25 cm). The fibula was inserted as a single strut in 29 patients and as a double-barrel construct in four patients. The reconstruction was augmented by a temporary uniplanar external fixator in 21 cases (all tibial reconstructions), by intramedullary nailing in two cases, and by bridge plating or mini plate fixation at both ends in 10 cases. A peroneal flap was simultaneously transferred to monitor the survival of the fibular flap in 29 cases. In the remaining four cases, ultrasonography was used to detect the patency of the vascular anastomosis. The average follow-up time was 53 months (range: 22-216 months).

The amount of hypertrophy was evaluated using the calculation method of Falder et al.^3^ and expressed as the difference between the original fibular width measured by the immediate postoperative X-ray and that in the final X-ray image. To avoid magnification error, all images were obtained from a standard distance, which was set to 1.5 m for the lower extremity and 1.2 m for the upper extremity. A picture archiving and communication system (PACS) was also employed to collect and measure the images used in this study. Two independent observers measured the maximum and minimum widths of the transferred fibula on the immediate post-operative X-ray films (AP and lateral views), and the average value of these four measurements was recorded as the original fibular width. This procedure was repeated on the final X-rays to obtain the final fibular width. The difference between these two values gave us the amount of hypertrophy that occurred during the follow-up period, which was expressed as a percentage.

### Statistical analysis

A number of statistical tests, including the Pearson correlation, Mann–Whitney *U*-test, one-way ANOVA, student *T* test, and paired samples *T* test, were used to investigate the presumed association between the quantitative and qualitative variables obtained from this study. The results were analyzed using the statistical program IBM SPSS Statistics Version 23 (IBM SPSS Corp, Armonk, NY, USA). A correlation analysis between hypertrophy and age, defect size, duration of follow up, and fibular length was performed to reveal any mathematical relationships between the listed variables. The significance level was set to *P* < 0.05.

## Results

Flap incorporation into the recipient bone was observed in all patients, including the two cases of avascular flaps. No statistically significant correlation among hypertrophy, age, defect size, duration of follow up, and fibular length was found; however, hypertrophy proved to be more pronounced in males than in females (*P* < 0.05).

All viable fibulas developed statistically significant, detectable radiological changes at their new location with a mean calculated hypertrophy of 74.8% (*P* < 0.05); however, individual values fluctuated in a very wide range between −4% (atrophy) and 214% (hypertrophy). The only cases without any positive values were those of the two avascular fibulas, which showed slight atrophy (negative values) at final evaluation. Objective hypertrophy, described as 20% or over by de Boer and Wood,^4^ was seen in only 19 lower extremity reconstructions, which were supplemented with temporary external fixation. A statistically significant difference in hypertrophy was detected between the upper and lower extremities in favor of the lower extremity (*P* < 0.05) (upper extremity average: 8.4%; lower extremity average: 99.8%). A similar difference was seen when reconstructions supplemented with permanent and temporary fixation devices were compared, with an apparent superiority of the patients treated with temporary implants; however, the majority of cases with temporary implants were complicated by a stress fracture of the flap during the consolidation process. Although this complication caused alignment failures to variable degrees, it induced abundant new bone production and hypertrophy at the reconstruction site.

**Figure 1. A, B.**
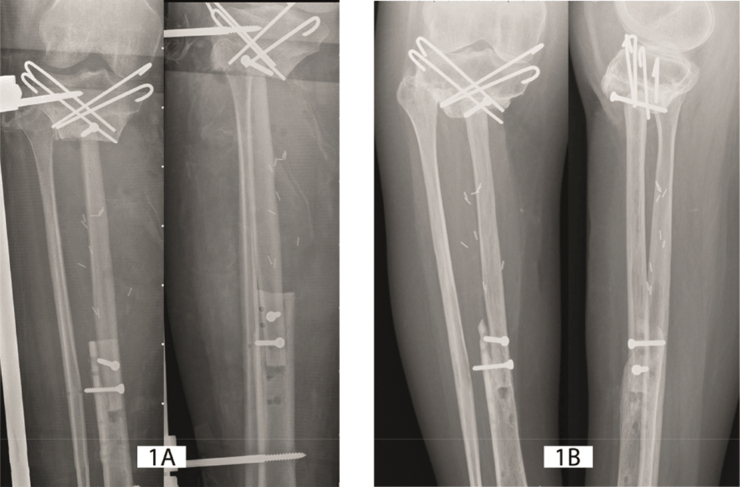
**A.** Early postoperative AP and lateral X-rays of a young female patient with tibial diaphyseal reconstruction following resection of a malignant bone tumor. This case was complicated by early postoperative arterial thrombosis, rendering the flap avascular. **B.** X-rays taken during the 26^th^ postoperative month reveal slight atrophy with diminished bone density at the flap, despite a successful union of the flap host interfaces.

When the bony consolidation of the 33 cases was examined in detail, four different types of flap hypertrophy were defined, with one mode including two subtypes. These types are further explained in detail as follows:

**Type 0.** The 1^st^ group comprises of the two avascular flaps, which showed slight atrophy despite a successful union at the flap host junction of both sides (Figure 1). Lack of hypertrophy suggested death of the transferred bone segment, which acted as a non-vascularized flap at the reconstruction site.^5^

**Figure 2. A-C.**
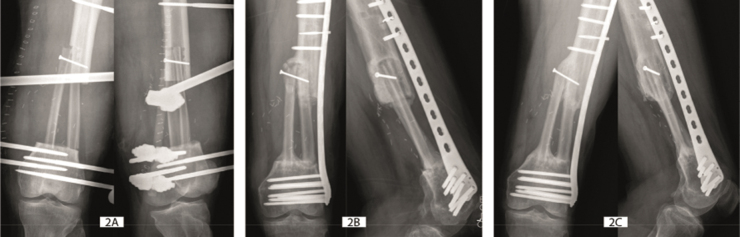
**A.** Double-barrel femoral reconstruction following resection of an osteomyelitic segment. **B.** Following an initial external fixation period of 6 weeks, stability was achieved with a permanent bridging plate. **C.** X-rays taken during the 27^th^ month after the initial operation reveal limited hypertrophy (type 1) due to stress shielding of the strong plate.

**Type 1.** This group is characterized by uniform limited thickening of the flap, with an average hypertrophy of 9.2% (range: 3.2-19). Although new bone production was detected in all patients, the average increase in diameter of the fibular flap did not exceed 20%, which was regarded as the minimum limit of objective hypertrophy.^4^ This group (12 cases) includes all of the reconstructions performed during upper- and lower-extremity reconstructions that were supported by permanent fixation (Figure 2).

**Figure 3. A-D.**
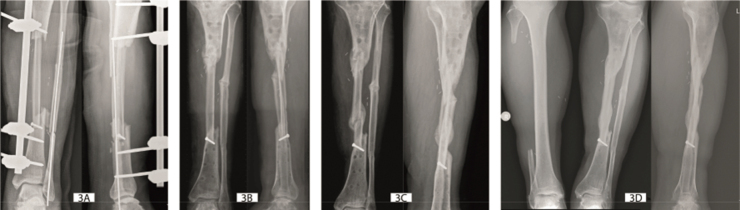
**A.** Tibial diaphyseal reconstruction following a crush injury due to a farm accident. **B.** X-rays taken during the 5^th^ postoperative month reveal a solid union. **C.** X-rays taken during the 8^th^ postoperative month reveal a healing stress fracture of the flap. **D.** X-rays taken during the 25^th^ postoperative month show hypertrophy of the entire flap, which is more pronounced at the fracture site.

**Type 2.** This type is characterized by abundant bone production triggered by a stress fracture of the flap and includes 11 of the 21 tibial reconstructions that were supplemented by temporary external fixation. Following flap union, all patients in this group developed an insufficiency fracture of the flap; this fracture incited a rapid and abundant new bone formation at the reconstruction site, reaching the girth of the host bone at the center and gradually expanding toward the edges of the fibular flap. This group is further divided into two subgroups based on the extent of the bony response:

**2a)** New bone formation was mainly localized at the fracture site and gradually decreasing toward the edges of the flap, resulting in fusiform hypertrophy (Figure 3). This type was seen in only in three patients.

**Figure 4. A-D.**
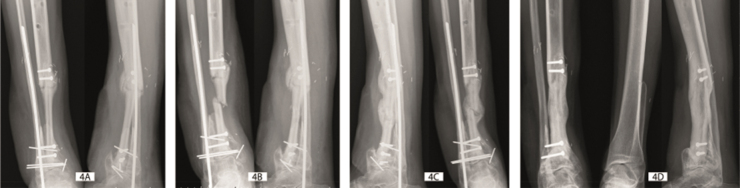
**A.** Reconstruction of distal tibia after a crush injury due to motorcycle accident. X-rays taken during the 4^th^ postoperative month reveal a bony union of the flap host interface. The patient wears a total contact brace to protect the slender fibular flap. **B.** X-rays taken during the 5^th^ postoperative month reveal a stress fracture of the flap. **C.** X-rays taken during the 7^th^ postoperative month depict a robust callus formation at the fracture site, triggering new periosteal bone formation throughout the fibular flap. **D.** X-rays taken during the 36^th^ postoperative month reveal massive bone hypertrophy, where the girth of the flap is slightly wider than that of the reconstructed bone.

**2b)** Bone production triggered by stress fracture was initially evident at the fracture site and spread rapidly to the entire fibula, resulting in a uniform tubular enlargement of the flap (Figure 4). The detected hypertrophy always exceeded 50% of the original width of the fibula, reaching the diameter of the host bone in the majority of cases.

**Figure 5. A-D.**
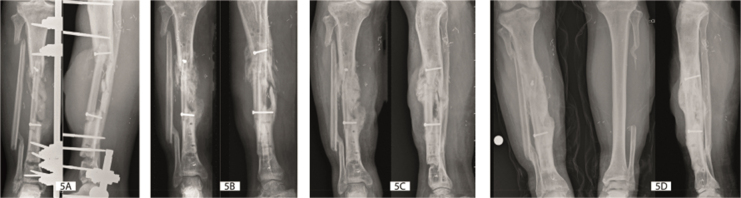
**A.** Tibial reconstruction using a vascularized fibular flap following a defected open fracture. Early postoperative images demonstrate new bone formation around the fibular flap, indicating a preserved periosteum at the reconstruction site. **B.** X-rays taken during the 5^th^ postoperative month reveal that the flap is united with the host bone, with gradual maturation of the peripheral bone wrapping, especially on the posterior and medial sides of the defect. **C.** X-rays taken during the 7^th^ postoperative month reveal a stress fracture of the flap, which facilitates new bone production at the flap’s anterior side. **D.** X-rays taken during the 35^th^ postoperative month show that this dual bone production generated massive hypertrophy, almost doubling the size of the reconstructed bone.

**Type 3.** This group included eight patients with traumatic defects of weight-bearing bones, where the enlarging fibular flap was surrounded by peripheral new bone formation and gradually integrated with it. A net increase in bone production was provided by hypertrophy of the fibular flap in addition to the enveloping soft tissue, which has osteogenic potential (Figure 5). This dual bone production caused the most prominent hypertrophy, with the diameter of hypertrophic bone exceeding the width of the recipient bone in seven cases. The fibular flap developed an insufficiency fracture in four cases, leading to gradual malalignment; however, the process of bone production did not seem to be negatively affected by this complication.

## Discussion

Although the ability of a vascularized fibular flap to develop hypertrophy has been previously noted by many authors, the variations observed in this process have not been analyzed or classified.^2,3,6,7^

The most objective method of showing hypertrophy of the bone seems to be to measure the thickness in mm and to show the net increase in the bone diameter in sequential X-ray images.^3,8^ The main problem with this method is the practical difficulty of determining the exact point for measurement. To overcome this problem, we measured the thinnest and thickest diameters of the flap from two views and used the average value of these four measurements. The amount of hypertrophy has been found to increase with time,^8^ but there is no consensus about the time of maturation of the hypertrophic process.^1,3,8^ Some authors have presented their results as early as 5-6 months postoperatively,^5,6^ with others stating that hypertrophy continues to evolve up to 2 years.^1,8^ In this study, 22 months is considered as the minimum follow-up time to refrain from evaluating premature hypertrophy.

Many variables are considered to affect the amount of hypertrophy; the initial requirement seems to be an adequate perfusion of the flap throughout the incorporation process. It has been observed that avascular flaps may unite with the host bone, but they do not show any signs of hypertrophy, which was seen in the two avascular fibular flaps in our series (Figure 1).^5^ Failure of hypertrophy demonstrates avascularity of the flap and is categorized as type 1 in our classification.

Mechanical stimulation due to the loading of the reconstructed segment is claimed to be the major factor determining the process of flap strengthening.^3,6,7,9–11^ In lower extremity reconstructions where the fibula does not act as a weight-bearing bone, and in cases with stress shielding of the fibula by a permanent implant, the amount of hypertrophy seems to be greatly restricted.^3,12^ The 2^nd^ type of hypertrophy in our group includes reconstructions in which hypertrophy is dwarfed by decreased mechanical stimulation. In the lower extremity, stress shielding by strong permanent implants seems to be the main reason for the limitation of hypertrophy. In the upper extremity, where mechanical loading of the reconstructed segment is very limited compared to the lower extremity, the average hypertrophy never reached the accepted limit of objective hypertrophy defined by de Boer and Wood (20% increase in diameter) in any of our cases.^4^ However, this observation should not be interpreted as failure of consolidation of the flap, since all patients in our series showed a thickening and apparent increase in the cortical density and ruffled external borders of the flap, indicating a hypertrophic state of bone. Rather, this finding could be interpreted as limited hypertrophy due to limited loading, since no stress fracture of the fibular flap was seen in this group and implant loosening was not observed in any case, proving that the amount of hypertrophy is capable of withstanding the stresses imparted to the flap without reaching the yield point of its strength. A similar observation was noted in double barrel reconstructions, where the centrally located inlay flap always hypertrophied; however, the peripherally located on lay flap showed limited hypertrophy or even atrophy during the consolidation process, proving that the lack of weight bearing eventually results in gradual bone resorption.^3,11^

Stress fracture of the flap is considered to be a complication^11^ and also seems to play a key role in the process of bone hypertrophy.^13–15^ Thirteen of the 21 tibial reconstructions that were supplemented with temporary external fixators in our series developed an insufficiency fracture at the fibular flap when stress shielding of the fixator was terminated; this event incited a rapid and robust response of new bone production, which, in addition to achieving solid bony union, resulted in marked hypertrophy of the flap, which reached the diameter of the recipient tibia in the majority of the patients. The hypertrophic process complicated by a fatigue fracture of the flap constitutes the 3^rd^ group (type 2) in our classification. The amount of fibular enlargement was greater than 50% in all patients, reaching 100% (doubling the diameter of the original fibula) in half of cases. The question of whether a fibular flap may show marked hypertrophy without developing a stress fracture has not yet been answered; in practice, we did not observe such a mode, so there is no such group in our classification system.

The role of the residual periosteum in new bone formation and flap hypertrophy has been subject to debate in the literature, since many authors have agreed that the best results are obtained in post-traumatic patients.^3^ Such an additional source of new bone formation may foster the process of hypertrophy, increasing the girth of the reconstructed segment and shortening the time of bony union. In eight of our cases with traumatic bone defects of the tibia with a partially preserved periosteum, this double source of bone production resulted in the most successful hypertrophy observed in this series, with an average enlargement of 170% compared with the original fibular width. This group formed the last type (type 3) in our hypertrophy classification.

Previous studies have concluded that the amount of hypertrophy is closely related to the age of the patient, as younger patients tend toward earlier and faster flap hypertrophy, which is probably related to their higher remodeling power and increased level of activity.^1,4,8^ Our results revealed no correlation between age and degree of hypertrophy or between bony gap and hypertrophy, supporting the results of Falder et al.^3^

The only reported classification of flap hypertrophy addressing vascularized fibular reconstructions in the literature is that proposed by De Boer et al.,^4^ which includes three sub-groups: periosteal bone formation (i.e., bone formation around the flap), endosteal bone formation, and a mixture of the two. We think that the proposed new classification addresses all the major variables thought to affect the outcome and helps predict the amount of hypertrophy and the possibility of a stress fracture.

De Boer et al.^4^ stated that due to its triangular cross section, subtle rotation of the fibula may give a false impression of hypertrophy of the fibula by as much as 20% in X-ray images. De Boer and Enneking et al. also stated that hypertrophy of up to 20% may occur in non-vascularized flaps and concluded that an irrefutable hypertrophy should be considered only in cases where the fibular width has increased to at least 20% greater than the original fibular width.^4,16^ We believe that such a limitation may result in underestimation of the hypertrophic process seen on the flaps when limited stresses are imparted to the fibular flap. Computer-assisted X-ray images enable us to visualize subtle changes occurring on the fibular flap and to perform precise measurements of the flap width during the consolidation process. Evaluation of high-quality computerized X-ray images revealed that lack of a flap response is seen only in avascular flaps, but hypertrophy beyond 20% is seen only in lower extremity reconstructions where the flap is not shielded by strong implants.

This study has demonstrated that the pattern of remodeling and hypertrophy did not follow an identical path in all vascularized fibular reconstructions. Rather, the amount of flap response following reconstruction varied depending on numerous variables affecting the consolidation process, from slight atrophy to massive bone formation beyond the extent of the host bone. An effort to classify the different modes of bone response may help us to understand the possible mechanisms of flap hypertrophy and enable us to predict the result, as well as complications such as stress fracture of the flap.

The patient group presented in this study exhibited reconstructions in all tubular bones of the extremities (radius, ulna, humerus, tibia, and femur) with all possible mechanisms of bone loss (trauma, tumor, and infection). This heterogeneity may be regarded as a limitation of the study. We believe that to detect different modes of flap hypertrophy, every possible variable must be included in the study group. Future studies comparing more uniform groups may help us to better clarify the presented modes of hypertrophy during flap maturation.

**Highlights**

- The long-term success of tubular bone reconstructions with vascularized fibular flaps are closely related to the process of flap hypertrophy.
- The process does not follow the same pattern in every reconstruction, and in this study we have described four different modes of flap hypertrophy based on known and unknown factors at the reconstruction site.
- Hypertrophy of the fibular flap seems to be closely related to adequate perfusion of the flap throughout the incorporation process, mechanical stimulation and stress shielding of the reconstruction site, the existence of a stress fracture, and the presence of any residual periosteum at the reconstruction site.

## Figures and Tables

**Table 1.** Detailed Data from the Enrolled Patients Concerning Hypertrophy, Reconstruction, and Fixation of the Affected Bone

Hypertrophy		Reconstruction		Reconstruction		Fixation	
Type	Number	Site	Number	Cause	Number	Type	Number
0	2	Femur	3	Trauma	17	Ex.Fix.	21
1	12	Tibia	21	Tumor	7	Plate	10
2A	3	Humerus	4	Nonunion	3	İMOS	2
2B	8	Ulna	3	Firearm	4		
3	8	Radius	2	Osteomyelitis	2		

Ex.Fix., External Fixator; IMOS, Intramedullary Osteosynthesis.

**Table 2.** Detailed Data Concerning Hypertrophy Type and Minimum, Maximum, and Average Values of Hypertrophy in the Fibular Flaps

Hypertrophy	Type 0	Type 1	Type 2A	Type 2B	Type 3
**Minimum (%)**	−4	+3.2	+52	+80	+77
**Maximum (%)**	−3	+19	+64	+154	+214
**Average (%)**	−3.5	+9.2%	+56.7	+104.6	+170
